# Anthocyanin Extract from Purple Sweet Potato Exacerbate Mitophagy to Ameliorate Pyroptosis in *Klebsiella pneumoniae* Infection

**DOI:** 10.3390/ijms222111422

**Published:** 2021-10-22

**Authors:** Guokai Dong, Nana Xu, Meng Wang, Yunyun Zhao, Fei Jiang, Huimin Bu, Jinjuan Liu, Bo Yuan, Rongpeng Li

**Affiliations:** 1 Key Laboratory of Biotechnology for Medicinal Plants of Jiangsu Province, School of Life Sciences, Jiangsu Normal University, Xuzhou 221116, China; dgkai@xzhmu.edu.cn (G.D.); xnn@xzhmu.edu.cn (N.X.); wm@xzhmu.edu.cn (M.W.); 2020190591@jsnu.edu.cn (Y.Z.); jiangf0319@jsnu.edu.cn (F.J.); buhuimin@xzhmu.edu.cn (H.B.); jjlbest@jsnu.edu.cn (J.L.); 2 Jiangsu Medical Engineering Research Center of Gene Detection and Department of Forensic Medicine, Xuzhou Medical University, Xuzhou 221004, China; 3 Laboratory of Morphology, School of Basic Medical Sciences, Xuzhou Medical University, Xuzhou 221004, China; 4 Department of Physiology, Xuzhou Medical University, Xuzhou 221004, China

**Keywords:** *Klebsiella pneumoniae*, purple sweet potato anthocyanins PSPAs, pyroptosis, mitophagy, NLRP3 inflammasome

## Abstract

Given the rise of morbidity and mortality caused by *Klebsiella pneumoniae* (KP), the increasing number of strains resistant to antibiotics, and the emergence of *hypervirulent Klebsiella pneumonia*, treatment of KP infection becomes difficult; thus, novel drugs are necessary for treatment. Anthocyanins, or natural flavonoids, have an extensive effect against bacterial infection. However, few studies on anti-KP are identified. Here, we evaluated the therapeutic effect of purple sweet potato anthocyanins (PSPAs) on KP, containing 98.7% delphinidin 3-sambubioside. Results showed that KP-infected mice after PSPAs treatment manifested decreased mortality, weakened lung injury, dampened inflammatory responses, and reduced bacterial systemic dissemination in vivo. In Vitro, PSPAs significantly suppressed pyroptosis and restricted NLRP3 inflammasome activation in alveolar macrophages infected with KP. As for the mechanism, PSPAs promote mitophagy by recruiting Parkin to the mitochondria. PSPAs-conferred mitophagy increased mitochondrial membrane potential and decreased mitochondrial reactive oxygen species and mitochondrial DNA, resulting in impaired NLRP3 inflammasome activation. In addition, the promotion of mitophagy by PSPAs required the Nrf2 signaling pathway. Collectively, these findings suggest that PSPAs are a potential option for the treatment of KP infection.

## 1. Introduction

*Klebsiella pneumoniae* (KP), singled out as an “urgent threat to human health”, is an opportunistic bacterial pathogen causing a variety of infectious diseases that are often difficult to treat, including pneumonia, bacteremia, urinary tract infections, and liver abscesses [1]. Recently, KP is gaining attention because of the rise of morbidity and mortality. In the United States, KP is considered as the common cause of hospital-acquired pneumonia, which accounts for 3% to 8% of all hospital bacterial infections [2], and the 30-day mortality of infection cases is as high as 39% [3]. In China, KP, which is composed of 11.9% of pathogens, is isolated from ventilator-associated pneumonia and ICU-acquired pneumonia [4]. Another feature of these infections is the increasing number of strains resistant to antibiotics. By one count, this pathogen accounts for 73.9% of carbapenem-resistant Enterobacteriaceae [5]. Furthermore, the emergence of *hypervirulent Klebsiella pneumoniae* and the spread of carbapenem-resistant *hypervirulent Klebsiella* colonization in ICU COVID-19 patients pose a great challenge to public health [6]. The emergence of resistant strains is bound to compromise the ability to cure KP infections with a general slowdown in the development and commercialization of novel antibiotics. Preventive vaccines and monoclonal antibodies represent novel approaches to fight KP. However, the great serotype variability requires either high-valency vaccines or monoclonal antibody cocktails to obtain acceptable coverage. The available vivo models are also a limitation because of the differences in KP pathogenicity between humans and animal models [7]. As such, new therapeutic agents are necessary to improve outcomes in patients with KP.

Regulation of the innate immune system to manipulate the course of infectious diseases was a novel attempt. Our previous findings demonstrated that autophagy-mediated bacterial clearance during *Pseudomonas aeruginosa* infection [8,9]. KP pathogenesis depends upon the interactions between the microbe and the host defense [10]. Pyroptosis is the innate immune response to the pathogen in infected host cells. Accumulating evidence indicates that pyroptosis in alveolar macrophages (AMs) is related to NLRP3 inflammasome, which is activated by invading of KP [11], and then induces the releasing of the secondary pro-inflammatory factors to participate in the elimination of invasive bacteria [12]. However, investigators found that pyroptosis induced by lethal toxin can lead to significant lung tissue injury [13]. It can be taken as that the amplified inflammation induced by the excessive release of pro-inflammatory factors aggravates the lung injury [14]. Other reliable evidence suggests that mitophagy can remove damaged mitochondria that are involved in promoting the activation of the NLRP3 inflammasome, and reduce the inflammatory response [15]. In addition, sepsis is more likely to cause tissue damage and death in PINK1 and Parkin, which is the main way to induce mitophagy, knockout mice [16]. Therefore, the regulation of pyroptosis and mitophagy may be a novel strategy for treating KP infection.

Medicinal plants have proven their value as sources with therapeutic potential in history, and many compounds from natural products have been used as drugs for the treatment of diverse indications, such as paclitaxel, galanthamine, and artemisinin. At present, plant-derived natural products, including quercetin, resveratrol, curcumin, and epigallocatechin-3-*O*-gallate, have been used in clinical trials related to anti-inflammatory mechanisms [17]. Extensive research has been conducted to find out the potent natural products that help to treat lung infections [18]. Therefore, natural products, particularly plants, represent a novel source of anti-microbial agents [19,20]. However, studies on mitophagy and pyroptosis as the therapeutic targets of natural products to defense against pathogen infections are rarely reported. Sweet potato (*Ipomoea batatas*) is a dicotyledonous plant, and belongs to the family of Convolvulaceae, which is a globally important food crop, and considered a valuable source of unique natural products, as rich in various phenolic and flavonoid bioactive compounds [21]. Purple sweet potato is a kind of sweet potato with purple flesh, possessing high anthocyanin content [21,22]. It is reported that the content of anthocyanin in purple sweet potatoes is similar to those of anthocyanins in crops with the highest yield, such as blueberries, blackberries, cranberries, and grapes [23]. Anthocyanins, which belong to flavonoids and primarily exist in the manner of glycosides, are considered as a natural compound with low toxicity and health care functions [24]. At present, growing evidence suggests that the use of anthocyanins has broad anti-inflammatory and anti-oxidant activity in infection, which might be associated with the activation of transcription factor NF-E2-related factor 2 (Nrf2) [25]. Notably, small molecule activators of Nrf2 support mitochondrial integrity by promoting mitophagy [26]. Purple sweet potato anthocyanins (PSPAs) are compounds of many varieties of anthocyanins, which have high heat resistance and ultraviolet stability. Accumulating evidence indicates that PSPAs have anti-inflammatory, anti-oxidant, anti-tumor, and other biological activities [23]. However, to date, the functions and mechanisms of the actions of PSPAs in pathogenic infection remain unknown, as well as whether mitophagy is promoted by the Nrf2 activation or not.

At present, the importance of mitophagy and pyroptosis in pulmonary bacterial infection has not been fully studied, and studies on mitophagy and pyroptosis as the therapeutic targets of natural products to defense against pathogen infections are rarely reported. The latest study showed that Epigallocatechin-3-gallate attenuated acute pancreatitis induced lung injury by targeting mitochondrial reactive oxygen species triggered NLRP3 inflammasome activation [27]. In another study, Sestrin 2 protected against LPS-induced acute lung injury by inducing mitophagy in AMs [28]. The above studies were not based on infection models, and the role of mitophagy in the inhibition of pyroptosis was not described in detail. Thus, further understanding of the molecular mechanisms of mitophagy and pyroptosis in KP infection is needed to identify potential more effective therapeutic targets. In this study, we extracted PSPAs from the root of purple sweet potato and studied their role in host defense against KP infection. Our objectives were to investigate the regulatory effects of PSPAs on mitophagy and pyroptosis in KP infection and to observe the role of the Nrf2 signal. Here, we hypothesized that PSPAs maintain mitochondrial homeostasis of AMs through mitophagy in KP infection. Our experimental data confirmed that PSPAs play a protective role in KP-infected AMs by promoting mitophagy and regulating pyroptosis through the Nrf2 signaling pathway.

## 2. Results

### 2.1. PSPAs Decreased Mortality Rates of KP–Infected Mice

**Figure 1:**
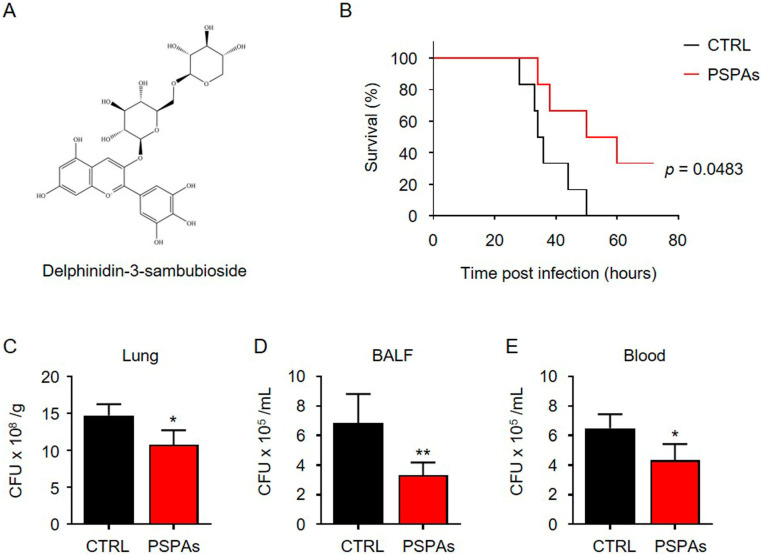
PSPAs decreased the mortality rates of KP-infected mice. (**A**) The structures of Delphinidin-3-sambubioside. (**B**) Mice were intraperitoneally injected with PSPAs (30 mg/kg mouse), daily, and after KP infection. Mice (*n* = 6) were infected with 1 × 10^7^ CFU KP/mouse through the nasal cavity. Kaplan–Meier survival curves were obtained (*p* = 0.0483; 95% confidence interval: 0.8594 to 12.4, log-rank test). 24 h after infection with 1 × 10^7^ CFU KP/mouse through the nasal cavity with or without 30 mg/L of PSPAs, bacterial loads in (**C**) lung homogenate, (**D**) BALF, and (**E**) blood were determined. Data (mean ± SEM) are representative of three independent experiments. One-way ANOVA (Tukey’s post hoc); * *p* < 0.05, ** *p* < 0.01.

We have purified an anthocyanin derivative, PSPAs, from the purple sweet potato Ningzishu 4, and found that the most abundant anthocyanins in the extract was Dp3-Sam with 98.7% of the total amount of the anthocyanins (Figure 1A), and Dp3-Sam has been proven to have anti-inflammatory properties in vivo and in vitro [29]. In evaluating the role of PSPAs in KP infection, KP (1.0 × 10^7^ CFU/mouse) was intranasally instilled in C57BL/6J mice to establish an acute pneumonia model, and PSPAs were administered to the mice by i.p. injection at a dose of 30 mg/kg body weight. For the control group, we only challenged mice with KP. By contrast, for the PSPAs-treated group, the mice were injected daily and after KP infection. The survival curves of these two groups of mice (six mice/group) were compared by Kaplan–Meier (Figure 1B), showing that PSPAs effectively decreased the mortality rates in KP-infected mice. Notably, all infected mice died at 50 h, whereas 50% of mice receiving PSPAs treatment remained alive during the entire period of observation. Meanwhile, we found that bacterial CFUs in the lungs and bronchoalveolar lavage fluid (BALF) of PSPAs-treated mice were significantly lower than that of control mice (Figure 1C,D). We detected the bacterial load in the blood to observe the diffusion of bacteria in the body. Consequently, the decrease was evident in the treatment of PSPAs (Figure 1E). These results suggested that PSPAs contributed to the host defense against KP in pneumonia models.

### 2.2. PSPAs Reduced Lung Injury and Inflammatory Responses in Mice Infected with KP

**Figure 2:**
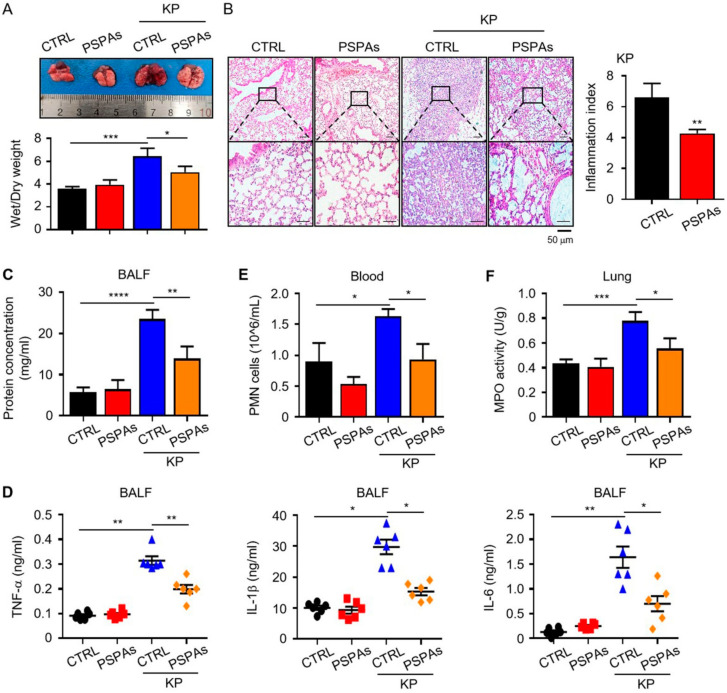
PSPAs reduce lung injury and inflammatory responses in mice infected with KP. PSPAs-treated mice and WT mice were infected with 1 × 10^7^ CFU KP for 24 h. (**A**) The mice lung images and the ratio of wet/dry weight were evaluated. (**B**) Representative histologic sections of lung tissues from mice of different groups were stained by hematoxylin and eosin (H&E), and a lung injury score was used. (**C**) The protein concentration in BALF was determined by bicinchoninic acid (BCA) protein quantification. (**D**) Inflammatory cytokines were measured by ELISA in the BALF of mice in different groups. (**E**) The number of neutrophils in the blood was counted by routine blood analyses. (**F**) The MPO activity of the lung was detected by a chemical chromatogram. Data (mean ± SEM) are representative of three independent experiments. One-way ANOVA (Tukey’s post hoc); * *p* < 0.05, ** *p* < 0.01, *** *p* < 0.001, **** *p* < 0.0001.

We confirmed the further effect of PSPAs against KP in vivo. First, we observed the gross morphological change (Figure 2A). The lungs of KP-infected mice became swollen and enlarged and displayed gross lesions with multifocal-to-coalescent dark-red discoloration in comparison with uninfected mice. However, PSPAs treatment mitigated such changes. In addition, we detected the ratio of lung wet/dry (W/D) weight of the lungs at 24 h post-infection and found that the W/D weight of mice treated with PSPAs decreased. Furthermore, the pathological changes of lung tissue were observed under a light microscope. Both of the infected lung tissues displayed lobular pneumonia, characterized by infiltration of inflammatory cells, destruction of the alveolar wall, alveolar hemorrhage, and dilated lung blood capillaries. Evidently, KP caused lung abscess and pulmonary consolidation, and PSPAs treatment reversed the changes (Figure 2B). The concentration of protein in BALF was measured to gauge the inflammatory responses after KP infection, which were significantly stronger compared with those in control mice but relatively low in PSPAs-treated mice (Figure 2C). The degree of inflammatory reaction in KP-infected mice was also lighter by detecting TNF-α, IL-1β, and IL-6 levels in BALF (Figure 2D) and the number of neutrophils in the blood (Figure 2E) in BALF after PSPAs treatment. In addition, we detected myeloperoxidase (MPO), which was released from polymorphonuclear neutrophils (PMN) in the lung tissue in response to exposure to various pulmonary insults and may serve as an indicator of the degree of infection. The MPO activity of the lung in PSPAs-treated mice was significantly lower than that of untreated infected mice (Figure 2F). Collectively, these data indicated that the severe lung injury and serious inflammation reaction caused by KP can be alleviated by PSPAs treatment, which can reduce the mortality of mice.

### 2.3. PSPAs Attenuated KP-Induced Pyroptsis in AMs

**Figure 3:**
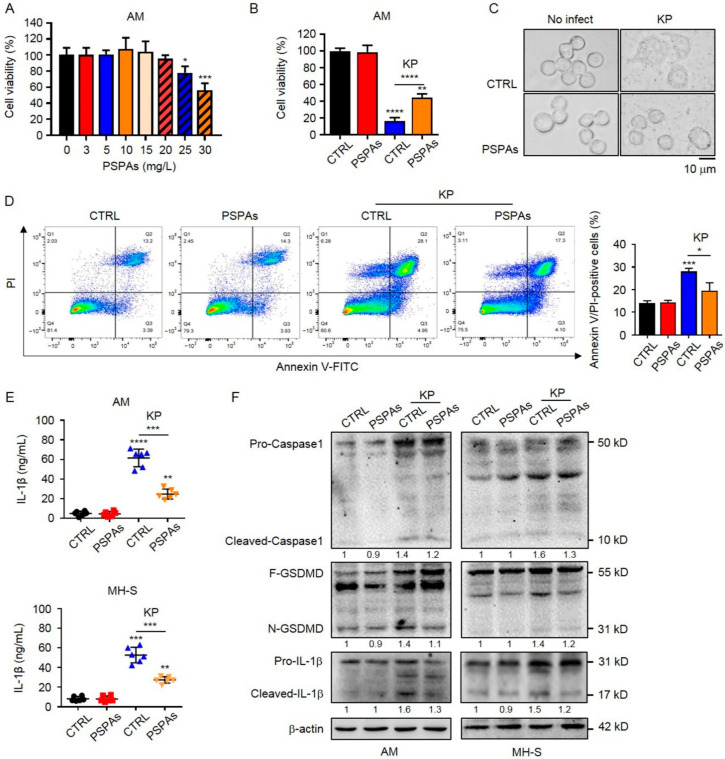
PSPAs attenuated KP-induced pyroptosis in AMs. (**A**) The effect of different concentrations of PSPAs on AMs for 24 h was determined by MTT assay. AMs treated with 5 mg/L PSPAs and WT AMs were infected with KP at a multiplicity of infection (MOI) of 20:1 for 6 h. (**B**) Viability of the cells was determined by MTT assay. (**C**) Morphological change of cells was observed under an inverted microscope. (**D**) Flow cytometry of propidium iodide (PI) and Annexin V-FITC-stained cells. (**E**) IL-1β in cell culture supernatant assessed by ELISA in AMs and MH-S treated as in (**B**). (**F**) Cells were lysed to measure Caspase1, GSDMD, and IL-1β levels by immunoblotting. Data (mean ± SEM) are representative of three independent experiments. One-way ANOVA (Tukey’s post hoc); * *p* < 0.05, ** *p* < 0.01, *** *p* < 0.001, **** *p* < 0.0001.

Several previous studies have shown that AMs critically contribute to pulmonary infection [30], and when AMs are “paralysis”, pneumonia caused by pathogens becomes severe [31]. We hypothesized that PSPAs could reduce the damage of KP to AMs. We first determined the safe dose of PSPAs and found that PSPAs did not inhibit the growth of AMs cells at 20 mg/L (Figure 3A). Therefore, 5 mg/L was selected as the safe concentration for subsequent experiments, and the survival of AMs cells with or without PSPAs after KP infection was detected by MTT. The results showed a significant increase in the viability of AMs cells treated with 5 mg/L of PSPAs after 24 h KP infection (Figure 3B). Meanwhile, after KP infection, AMs cells exhibited cell swelling and the rupture of the membrane, which was a signature morphology of pyroptosis (Figure 3C). However, the above-mentioned morphological changes were alleviated after treatment with PSPAs. Pyroptosis is an inflammatory programmed cell death associated with infection, and previous research has shown that pyroptosis occurs in AMs during LPS-induced acute lung injury in mice [14]. We hypothesized that pyroptosis had the same effect on KP infection. We detected the percentage of annexin V-FITC and PI-positive cells by flow cytometry assay, which indicated pyroptosis. Consequently, the percentage was significantly upregulated in KP-infected AMs cells and reduced following PSPAs treatment (Figure 3D). The elevated level of IL-1β is another sign of pyroptosis. Next, we measured the levels of IL-1β in the supernatant of AMs and MH-S by ELISA. As shown in Figure 3E, the levels of IL-1β induced by KP were reduced by PSPAs. Then, pyroptosis-associated proteins were examined by Western blotting. In KP-infected AMs and MH-S, the protein expression of Cleaved-caspase-1, GSDMD-N, and Cleaved-IL-1β was highly increased, whereas PSPAs inhibited the protein expressions induced by KP (Figure 3F). Collectively, these results suggested that the overly active inflammatory response and severe lung injury induced by KP were partly caused by pyroptosis, and PSPAs significantly attenuated pyroptosis in KP-infected AMs cells.

### 2.4. PSPAs Suppresses NLRP3 Inflammasome Activation and Mitochondrial Dysfunction Induced by KP

**Figure 4:**
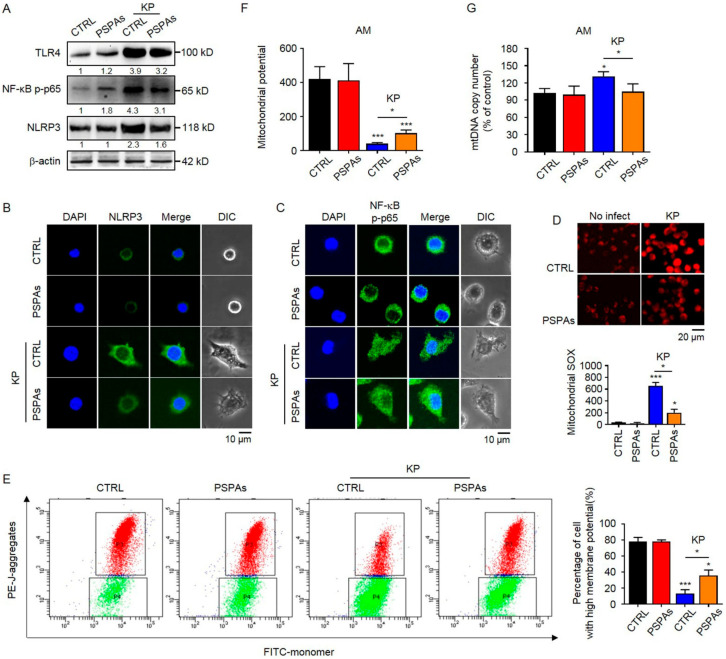
PSPAs suppresses NLRP3 inflammasome activation and mitochondrial dysfunction induced by KP. (**A**) Immunoblotting of TLR4 and NLRP3 and phosphorylation of NF-κB p-p65 in PSPAs-treated and controlled AMs after treating with or without KP at an MOI of 20:1 for 6 h. Representative images of (**B**) NLRP3 (green) and (**C**) NF-κB p-p65 (green) and DAPI (blue).NF-κB p-p65 by laser confocal microscopy. (**D**) MitoSOX Red Mitochondrial Superoxide Indicator detected by fluorescence assay and mitochondrial potential measured by (**E**) JC-1 flow cytometry and (**F**) fluorescence assay. (**G**) Mitochondrial DNA copies were analyzed by qRT-PCR. Data (mean ± SEM) are representative of three independent experiments. One-way ANOVA (Tukey’s post-hoc); * *p* < 0.05, *** *p* < 0.001.

Activation of the NLRP3 inflammasome is essential for KP-induced AMs pyroptosis [32]. However, activation of the canonical NLRP3 inflammasome requires two signals. One is the NF-κB-dependent priming signal that leads to the expression of pro-IL-1β and NLRP3, and the other one is a cytosolic stress signal that triggers the assembly of the NLRP3 inflammasome. Then, we investigated whether PSPAs inhibited NLRP3 inflammasome activation. The WB results showed that the protein levels of TLR4, NF-κB p-p65, and NLRP3 increased in KP-infected AMs, and PSPAs treatment reduced the increased protein levels (Figure 4A). The inhibition of NLRP3 by PSPAs was also confirmed using confocal laser-scanning microscopy (Figure 4B and Figure S1A). In addition, we observed that KP promoted NF-κB p-p65 nuclear translocation, whereas treatment with PSPAs resulted in the reversal of this process (Figure 4C and Figure S1B). Based on previous reports, mitochondrial dysfunctions played an important role in the activation of NLRP3 [33]. Thus, we measured mitochondrial membrane potential (MMP) by JC-1 flow cytometry and fluorescence assay and mitochondrial reactive oxygen species (mtROS) generation by fluorescence assay using MitoSOX as described to address the mitochondrial functional alterations. The results showed that the mitochondria in KP-infected cells produced more mtROS than the control cells, and a significant reduction was observed after PSPAs treatment (Figure 4D). Compared with the control group, KP-infected cells also showed a decline in MMP as determined by the uptake of JC-1, which was recovered by PSPAs treatment (Figure 4E,F). In addition, similar to elevated mtROS, elevated Mitochondrial DNA (mtDNA) release was correlated with the accumulation of damaged mitochondria, which promoted the activation of NLRP3 inflammasome [34]. We measured the levels of mtDNA content and found that PSPAs treatment resulted in decreased mtDNA copy number increased by KP (Figure 4G). Thus, these results indicated that KP impaired mitochondrial function and induced NLRP3 inflammasome activation, whereas all aforementioned changes were substantially mitigated by PSPAs treatment.

### 2.5. PSPAs Ameliorates Mitochondrial Dysfunction through Mitophagy to Inhibit KP-Induced Pyroptosis

**Figure 5:**
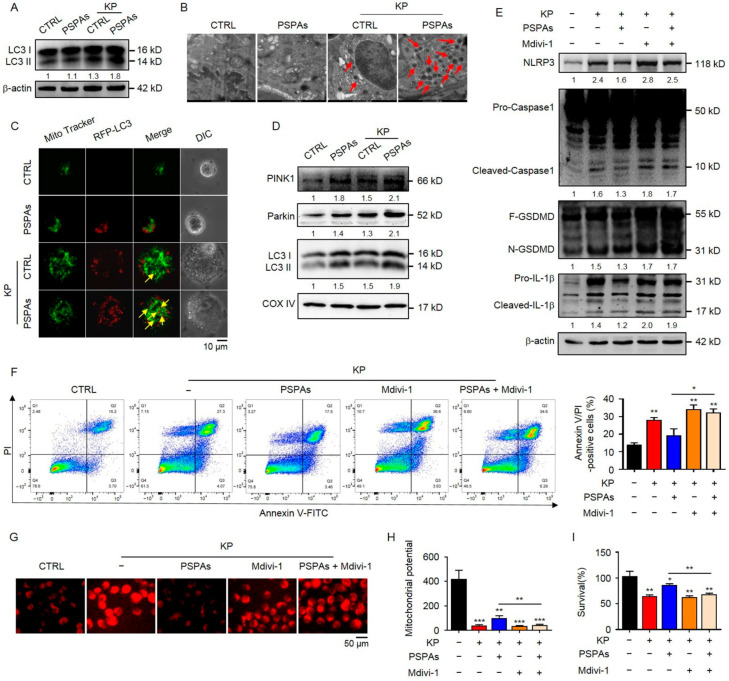
PSPAs ameliorate mitochondrial dysfunction through mitophagy to inhibit KP-induced pyroptosis. (**A**) Immunoblotting of LC3 in PSPAs-treated and controlled AMs after treating with or without KP at an MOI of 20:1 for 6 h. (**B**) Autophagosome images of cells treated as in (**A**) under a transmission electron microscope. Red arrows indicate autophagosomes. (**C**) Cells were transfected with RFP-LC3 plasmids for 24 h, followed by treatment with or without KP at an MOI of 20:1 for 6 h and staining with MitoTracker Green for 30 min. The number of co-localized RFP-LC3 puncta (red) and MitoTracker Green (green) was quantified. Yellow arrows indicate colocalization. (**D**) Immunoblotting of Parkin, PINK1, and LC3 in the mitochondrion of cells treated as in (**A**). (**E**) Immunoblotting of NLRP3, Caspase1, GSDMD, and IL-1β in PSPAs-treated and controlled AMs treated with or without KP at an MOI of 20:1 for 6 h, in combination with or without 2 μM Mdivi-1. (**F**) Flow cytometric analysis of the Annexin V/PI positive rate, (**G**) MtROS, and (**H**) JC-1 detected by fluorescence assay in cells treated as in (**E**). (**I**) MTT assay of PSPAs-treated and controlled AMs with or without KP at an MOI of 20:1 for 6 h, in combination with or without 1 μM Mdivi-1. Data (mean ± SEM) are representative of three independent experiments. One-way ANOVA (Tukey’s post-hoc); * *p* < 0.05, ** *p* < 0.01, *** *p* < 0.001.

Mitophagy, selective autophagy of the mitochondria, is an important form of mitochondrial quality control that eliminates damaged mitochondria [35]. We hypothesized that PSPAs stimulated mitophagy in AMs to remove the damaged mitochondria. In testing this hypothesis, we first investigated the change of LC3, which is a marker of autophagy. We found that PSPAs promoted the conversion of LC3-I to lipidated LC3-II in KP-infected AMs cells through the WB experiment (Figure 5A), and the formation of autophagosomes, as direct evidence, was observed by transmission electron microscopic imaging in AMs cells (Figure 5B and Figure S1C). Further research found that significantly increased co-localization of the autophagosome with the mitochondria was induced by PSPAs in KP-infected AMs cells, as evidenced by the merged fluorescent signaling of RFP-LC3 and MitoTracker (Figure 5C and Figure S1D), indicating that the mitochondria were engulfed by an autophagosome. Previous studies have identified the PINK1/Parkin pathway as a key signaling pathway that mediates mitophagy in mammalian cells, and the translocation of Parkin to the mitochondria indicates mitophagy [36]. We confirmed the increased expression of PINK1, Parkin, and LC3-II in the mitochondria by immunoblotting (Figure 5D), suggesting that mitophagy promoted by PSPAs was related to mitochondrial Parkin translocation. We treated PSPAs-treated and controlled AMs cells with Mdivi-1 to inhibit mitophagy after KP infection and investigate whether PSPAs inhibited the formation of NLRP3 inflammasome and pyroptosis via mitophagy in AMs. The result of WB showed that protein expression of Cleaved-caspase-1, GSDMD-N, and Cleaved-IL-1β relieved by PSPAs was recovered after Mdivi-1 treatment (Figure 5E), and Annexin V/PI-positive cells, measured by flow cytometry assay, also increased correspondingly (Figure 5F). We also found that mtROS relieved by PSPAs were recovered after Mdivi-1 treatment (Figure 5G and Figure S1E), and the alterations of MMP were similar to that of mtROS (Figure 5H). Furthermore, Mdivi-1 treatment reversed PSPAs’ increased cell survival (Figure 5I). Collectively, these data indicate that the protective mechanism of PSPAs against infection is to promote mitophagy to ameliorate KP-induced mitochondrial dysfunction and then inhibit the formation of NLRP3 inflammasome and pyroptosis.

### 2.6. Nrf2 Is Required in PSPAs-Induced Mitophagy in AMs

**Figure 6:**
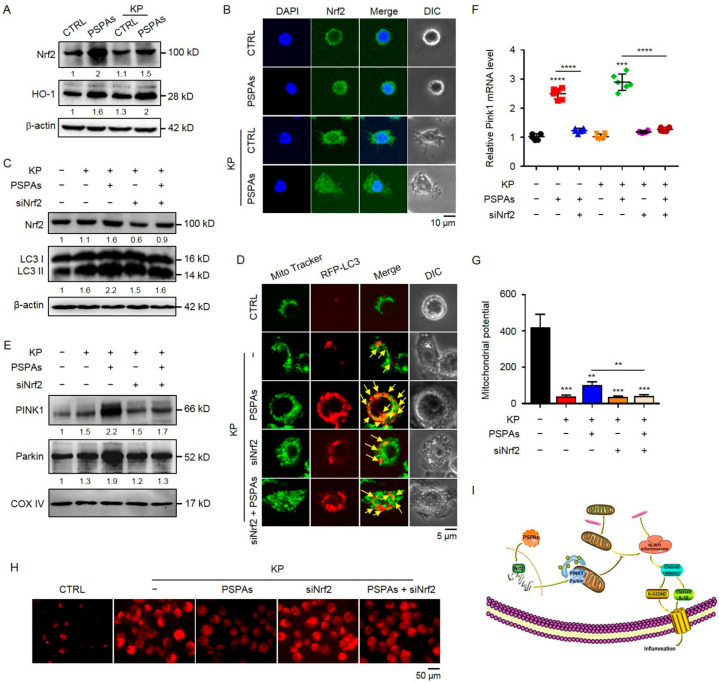
Nrf2 is required in PSPAs-induced mitophagy in AMs. (**A**) Immunoblotting of Nrf2 and HO-1 in PSPAs-treated and controlled AMs after treating with or without KP at an MOI of 20:1 for 6 h. (**B**) Cells were stained with Nrf2 antibody (green) and DAPI (blue). The nuclear translocation of Nrf2 was assessed by immunofluorescence staining in cells treated as in (**A**). Nrf2-silenced and AMs-controlled cells were infected by KP with or without PSPAs for 6 h. (**C**) The levels of Nrf2 and LC3 were determined by immunoblotting, and (**D**) the colocalization of MitoTracker Green (green) and RFP-LC3 puncta (red) was observed by confocal microscopy. Yellow arrows indicate colocalization (**E**) Parkin and PINK1 in the mitochondrial fractions were detected by immunoblotting in cells treated as in (**C**). (**F**) The mRNA levels of PINK1 were measured by qRT-PCR in Nrf2-silenced and AMs-controlled cells infected by KP with or without PSPAs. (**G**) JC-1 and (**H**) MtROS were detected by fluorescence assay in cells treated as in (**C**). (**I**) Diagram delineating a pathway in PSPAs against KP infection. PSPAs activate the Nrf2 signaling pathway and exacerbate mitophagy to attenuate KB-induced pyroptosis through mitophagy. Data (mean ± SEM) are representative of three independent experiments. One-way ANOVA (Tukey’s post-hoc); ** *p* < 0.01, *** *p* < 0.001, **** *p* < 0.0001.

Nrf2, encoded by the nfe2l2 gene, is a transcription factor, orchestrating the expression of cytoprotective genes to maintain homeostasis, which can be activated by anthocyanins [37]. Recent evidence has shown that Nrf2 mediates a mitochondria-protective signal and regulates autophagy [38]. Here, we investigated the role of the Nrf2 pathway in PSPAs-promoted mitophagy. We first detected whether the Nrf2 pathway can be activated by PSPAs. The results of WB showed that PSPAs promoted the expression of Nrf2 and hemeoxygenase-1(HO-1) in AMs cells (Figure 6A), and the latter was primarily regulated by Nrf2. Notably, in the case of KP infection, we found that PSPAs promoted Nrf2 to enter into the nucleus under a confocal laser-scanning microscope (Figure 6B and Figure S1F). These results showed that PSPAs can activate the Nrf2 pathway, and this effect was significantly enhanced in the case of infection. We introduced Nrf2-interfering RNA into AMs cells to block Nrf2 expression and investigate whether Nrf2 was a potential trigger in PSPAs-mediated mitophagy. Our results showed that PSPAs-induced accumulation of LC3-II of Nrf2-silenced AMs cells was decreased after KP infection (Figure 6C). Consequently, after the knockdown of Nrf2, the co-localization of RFP-LC3 with MitoTracker was inhibited under a confocal laser-scanning microscope (Figure 6D and Figure S1G), and the PSPAs-induced Parkin accumulation in the mitochondrial fraction was abolished through WB experiment (Figure 6E). In addition, the PINK1 mRNA level was significantly reduced by qRT-PCR (Figure 6F). Moreover, we found that Nrf2 knockdown attenuated the protective effects of PSPAs on maintaining MMP (Figure 6G) and reducing mtROS production (Figure 6H and Figure S1H). Collectively, these results verified that PSPAs induced mitophagy-mediated protection of mitochondrial function through the Nrf2 signaling pathway in KP-infected AMs cells (Figure 6I).

## 3. Discussion

In this study, we demonstrated that PSPAs had a protective role in lung inflammation induced by KP and revealed the mechanism of action. We observed a milder disease phenotype in PSPAs-treated mice in our experiments, which included increased survival, decreased inflammatory response, and slight lung injury, compared with WT mice (Figure 1 and Figure 2). Meanwhile, our data suggested that PSPAs alleviated pyroptosis in KP-infected AMs (Figure 3), indicating that the protective effect of PSPAs was associated with the inhibition of AMs pyroptosis caused by KP. Additional data demonstrated that the inhibition of pyroptosis was related to PSPAs-promoted mitophagy, which resulted in the reduction of NLRP3 inflammasome in KP-infected AMs (Figure 4 and Figure 5). We also found that PSPAs triggered Parkin mitochondrial translocation and beneficial mitophagy through the Nrf2 signaling pathway, resulting in the degradation of the mitochondria (Figure 6).

Our study proved the effectiveness of PSPAs against bacterial infection. As reported previously, anthocyanins were recognized as phyto-anti-inflammatory agents [39], and the role of PSPAs in the anti-inflammatory process has also been confirmed [23]. However, relative studies have focused on models of chronic noncommunicable diseases, such as cardiovascular diseases and diabetes, and few research has been conducted on acute infection models [40]. Little research is based on LPS-induced inflammation and even sepsis mouse models, and the contents are limited to phenotype and NF-κB and AP-1 signal transduction pathways. Other studies reported that protein-bound anthocyanin compounds from purple sweet potato reduced the expression of inducible nitric oxide synthases and tumor necrosis factor-α (TNF-α) in RAW264.7 cells stimulated by LPS [41], and Cyanidin-3-*O*-glucoside attenuated acute lung injury in sepsis rats through suppressing the NF-κB signaling pathway [42]. Our results were similar to previous observations that anthocyanins had an effect against bacterial infection. However, we used KP-induced infection models, and the main component of our extract was Dp3-Sam from purple sweet potato. In addition, we investigated the molecular mechanisms involved in the regulation of mitochondrial homeostasis in response to anthocyanins treatment in protecting against pathogenic infection. These findings illustrate the effect of anthocyanins against bacterial infection in more detail, providing a basis for the development of anthocyanins and purple sweet potato.

For the first time, we showed that PSPAs could inhibit pyroptosis in KP-infected AMs, and the suppressed pyroptosis was advantageous for host defense against KP infection. We demonstrated that KP-induced expression of Cleaved-caspase-1, GSDMD-N, and Cleaved-IL-1β was inhibited, and the percentage of annexin V-FITC and PI-positive cells upregulated in KP-infected AMs cells was reduced following PSPAs treatment (Figure 3D–F). Moreover, our study had shown that the bacterial CFUs in the lungs, BALF, and blood of PSPAs-treated mice, which were significantly lower than that of control mice (Figure 1C–E). Previous studies focused on the relationship between pyroptosis and injury. For instance, Piperine protects macrophages from pyroptosis and reduces IL-1β and HMGB1 release in the mouse model intraperitoneally infected with Escherichia coli [43]. Quercetin prevents neuronal injury via suppressing NLRP3 inflammasome and related pyroptosis [44]. Curcumin inhibits the activation of NLRP3 inflammasome-dependent pyroptosis through the upregulation of SIRT1 [45]. It has also been suggested that LPS-induced lung injury could be attenuated by inhibiting pyroptosis [14,28]. In addition to the attenuated lung injury, in this thesis, our study explored the problem that PSPAs regulated the innate immune system through pyroptosis. Pyroptosis, different from apoptosis, autophagy, and anoikis, is a pro-inflammatory death mode (membrane permeabilizing) with necrotic morphological characteristics, which is the innate immune response to the pathogen in infected host cells, and it is an important regulated cell death in KP-infected macrophage [12,46]. However, KP, as a facultative intracellular pathogen, induces necrosis (membrane permeabilizing) in bacteria-burdened cells to promote the release of bacteria, infect new cells, and drive further immune cell recruitment and tissue damage. Therefore, pharmacologic suppression of membrane-permeabilizing cell death during such infections may be a therapeutic strategy against KP [46]. Our study has shown that inhibition of pyroptosis limited the transmission of KP, indicating that the mechanism of fighting off pathogenic infection may be related to the regulation of host innate immunity in KP infection. From this perspective, addressing the treatment difficulty of increasing antibiotic resistance of pathogens is possible.

Another remarkable finding of the present study is that our study revealed the role of mitophagy in PSPAs against KP infection, inhibiting NLRP3 inflammasome activation and pyroptosis. Other reports and our study show that KP-induced pyroptosis requires NLRP3 inflammasome [11]. Mitophagy, which is the selective autophagy of mitochondria that removes unwanted or damaged mitochondria, is considered a key factor in the negative regulation of NLRP3 inflammasome activation [47]. In models related to inflammation such as alcoholic liver disease and high-fat diet, previous studies demonstrated that anthocyanins could inhibit NLRP3 inflammasome activation [48,49], but the role of mitophagy of immune cells was not discussed. In this study, we observed mitochondrial dysfunction (homeostatic disruption) in KP-infected AMs cells, exhibited by increased mtROS (Figure 4D) and mtDNA content (Figure 4G), and a decreased MMP (Figure 4E,F). In addition, PSPAs treatment rescued the KP-impaired mitochondrial homeostasis, and the activation of NLRP3 inflammasome was ameliorated correspondingly (Figure 4A,B). Further studies showed the key mechanism of PSPAs-mediated protective effects, which promoted mitochondrial Parkin translocation and mitophagy (Figure 5A–D). These results were similar to those of previous studies on the regulation of NLRP3 inflammasome activation by mitophagy in other natural products. For instance, Quercetin hinders microglial activation to alleviate neurotoxicity via the interplay between NLRP3 inflammasome and mitophagy [44], and Berberine suppresses influenza virus-triggered NLRP3 inflammasome activation in macrophages by inducing mitophagy and decreasing mitochondrial ROS [50]. However, our data highlighted the inhibition of pyroptosis by mitophagy during bacterial infection based on that (Figure 5E,F), providing new evidence supporting that mitophagy participates in the innate immune response. Meanwhile, we found that PSPAs inhibited KP-induced NF-κB, which was the first sign of NLRP3 activation. In particular, PSPAs alone slightly promoted NF-κB p-p65, which was inconsistent with other studies [51]. This finding may be related to the complexity of NF-κB signaling, which interacted directly or indirectly with many other signaling pathways, particularly in macrophages [52]. In addition, we found that mitophagy increased after only KP treatment. The compensatory mechanism of AMs in response to KP was also considered, providing new evidence for the involvement of mitophagy in the regulation of the innate immune system.

In our study, we determined that PSPAs promoted mitophagy through the Nrf2 pathway. Nrf2, a core transcription factor that maintains mitochondrial homeostasis, has been developed as an important drug target [26,53]. Recently, Nrf2 is considered a molecular target for sepsis patients in critical care to regulate the imbalance between pro-inflammatory and anti-inflammatory mechanisms [54]. Here, we found that PSPAs treatment could increase Nrf2 expression and nuclear translocation (Figure 6A,B), and knock-down of Nrf2 aggravates KP-mediated mitochondrial dysfunction (Figure 6G,H) by impeding PINK1 accumulation and Parkin recruitment on the mitochondrial membrane (Figure 6D,E) in response to PSPAs treatment, suggesting that Nrf2 plays a critical role in PSPAs in promoting mitophagy in KP-infected AMs cells. In line with our findings, small-molecule activators of Nrf2 support mitochondrial integrity by promoting mitophagy and conferring resistance to oxidative stress to relieve tissue and organ damage in research related to anti-inflammatory and antioxidant activities [55]. However, our study confirmed the role of the Nrf2 signaling pathway in the effect of anthocyanins against bacterial infection, which provided a basis for the application of PSPAs in other infection models. PSPAs have the potential for drug development for Nrf2.

Collectively, the important protective effect of anthocyanins from purple sweet potato against KP infection was reported, which was not reported by other studies. The substance promoted mitophagy through the Nrf2 signaling pathway and inhibited AMs pyroptosis caused by KP (Figure 6I). These results imply that PSPAs are a potential therapeutic agent against KP, providing an important theoretical basis for clinical research.

## 4. Material and Methods

### 4.1. Mice

C57BL/6J female mice (6–8 weeks) were obtained from Xipuer-BiKai Experimental Animals Co., Ltd. (Shanghai, China). All animal studies were approved by the Xuzhou Medical University Institutional Animal Care and Treatment Committee and performed in accordance with the animal care and institutional guidelines. The animal experimental procedures, including treatment, care, and endpoint choice, were in accordance with the “Animal Research: Reporting In Vivo Experiments” guidelines. Animal experiments were performed with randomization.

### 4.2. Primary Cells and Cell Lines

As mice were sacrificed, the thoracic cavity and trachea were exposed. A small incision was made in the trachea via a 1 mL syringe with an angiocath, and catheters were secured in place using a 6–0 silk suture. After lavaging three times with 1 mL of normal saline containing 1% FBS (PAN-Biotech, Aidenbach, Germany), the retained BALF was centrifuged at 600× *g*, 4 °C for 5 min. The cells were resuspended in RPMI 1640 medium (Life Technologies, Carlsbad, CA, USA) with 10% FBS and incubated at 37 °C in 5% CO_2_ (Xuzhou Special Gas Co., Xuzhou, China) for 1 h. When the macrophages adhered to the bottom surface, nonadherent cells were removed by washing with normal saline. MH-S murine AMs were obtained from American Type Culture Collection (Manassas, VA, USA). All cells were maintained in RPMI 1640 medium, supplemented with 10% FBS, 100 U/mL of penicillin (Thermo Fisher Scientific, Waltham, MA, USA), and 100 U/mL of streptomycin (Thermo Fisher Scientific, Waltham, MA, USA) in a humidified incubator at 37 °C in 5% CO_2_ atmosphere [9].

### 4.3. Bacteria Preparation and Infection Experiments

*Klebsiella pneumoniae* was provided by Dr. V. Miller (University of North Carolina). Bacteria were grown overnight in 5 mL of lysogeny broth (LB) at 37 °C and 220 rpm with shaking and pelleted by centrifugation at 5000× *g*. AMs and MH-S cells were changed to antibiotic-free medium and infected with bacteria an MOI of 20. Mice were anesthetized with 45 mg/kg of ketamine and instilled intranasally with 0.5 × 10^7^ CFU KP in 50 μL of PBS (*n* = 6). The symptoms of the mice were observed, and the mice were euthanized when they were moribund [56].

### 4.4. Histological Analysis

The extracted lung tissues of three independent mice were immersed in 4% paraformaldehyde (Sigma-Aldrich, Saint Louis, MO, USA) until they were completely fixed. Routine paraffin slices were prepared and stained by hematoxylin and eosin H&E. Then, the pathological features of lung tissues after infection were observed under a light microscope [56].

### 4.5. Inflammatory Cytokine Profiling

Cytokine concentrations of TNF-α, IL-1β, and IL-6 were measured using ELISA kits (BOSTER, Wuhan, China) in BALF and cell culture supernatant collected at the indicated times post-infection. The samples were collected, and 100 mL of aliquots were added to the coated microtiter wells. The cytokine concentrations were measured with corresponding horseradish peroxidase (HRP)-conjugated Abs. The values were read at 450 nm using a microplate reader (Thermo Fisher Scientific, Waltham, MA, USA) [56].

### 4.6. The Protein Concentration of Lung Lavage Samples

The protein concentration in BALF was measured. BALF were collected at the indicated times post-infection and determined via a BCA (KeyGen Biotech, Nanjing, China) assay, and absorbance was measured at 562 nm using a microplate reader (Thermo Fisher Scientific, Waltham, MA, USA) [57].

### 4.7. Lung W/D Weight Ratio

The lung W/D ratio was used to calculate pulmonary edema. The left lung of three independent mice was weighed immediately after collection as the wet weight and reweighed after being dried in an oven at 80 °C for 24 h as the dry weight. The W/D ratio was calculated as the wet weight divided by the dry weight [57].

### 4.8. MPO Activity Assay

Contents of MPO in lung tissue of three independent mice were measured using an MPO kit (Nanjing Jiancheng Bioengineering Institute, Nanjing, China). Two hundred microliters of the sample was added to each well, and after incubation for 30 min at 37 °C, 200 μL of bio-anti mouse MPO was added and incubated at 37 °C for 30 min. Afterward, ABS working solution and TMB color developing agent were added and incubated at 37 °C for 30 min in the dark. Color development solution and stop solution were added to each well, and the plate was read at 460 nm using a microplate reader (Thermo Fisher Scientific, Waltham, MA, USA) [58].

### 4.9. Bacterial Burden Assay

BALF, blood, and lung tissues were homogenized with normal saline (CR Double-Crane Pharmaceuticals Co., Ltd., Beijing, China) and spread on LB dishes to identify the bacteria. The dishes were cultured in a 37 °C incubator overnight, and colonies were counted. Duplicates were performed for each sample and control [9].

### 4.10. Polymorphonuclear Cell Counts in Peripheral Blood 

Mice were treated as previously described, and blood samples were collected from each animal for PMN cell counts by a Veterinary Animal Blood Counter (Mindray Biomedical Electronics Co., Shenzhen, China) as previously described [59].

### 4.11. Transfection of Small Interfering RNA, Plasmids, and Inhibitors

Nrf2 and scrambled small interfering RNAs were obtained from Santa Cruz Biotechnology (Santa Cruz, CA, USA). AMs cells were transfected with small interfering RNA (siRNA; 5 pM) and LC3-RFP G120A (100 ng) plasmids using Lipofectamine 2000 (Life Technologies, Carlsbad, CA, USA) for 24 h, following the manufacturer’s instructions [55]. In some cases, AMs cells were treated with a 2 μM mitophagy inhibitor (Mdivi-1, Sigma-Aldrich, Saint Louis, MO, USA) for 1 h before and during KP infection as indicated [60].

### 4.12. RNA Isolation and Quantitative Real-Time PCR

RNA was isolated from primary AMs using TRIzol (Life Technologies, Carlsbad, CA, USA). For first-strand cDNA synthesis, a total of 50 ng of DNA-free RNA was prepared and performed using the RevertAid First Strand cDNA Synthesis Kit (Thermo Fisher Scientific, Waltham, MA, USA). Primers for quantitative real-time PCR are shown in Table 1. The qRT-PCR assay was manipulated using iTag Universal SYBR Green Supermix (Bio-Rad, Hercules, CA, USA) in a CFX Connect Real-Time PCR Detection System (Bio-Rad), and the results were calculated through the 2^2DDCt^ threshold methodology following normalization with GAPDH [61].

### 4.13. mtDNA Content

Total DNA was isolated by standard phenol (Sangon Biotech, Shanghai, China) -chloroform (Lingfeng Chemical Reagent Co., Ltd., Shanghai, China) extraction. Fragments of the mitochondrial Nd2 gene and GAPDH gene were amplified in duplicate by qRT-PCR. Primers are shown in Table 1. MtDNA copy number was expressed as the mtDNA-to-nuclear-DNA ratio (Nd2 mtDNA/GAPDH DNA) [55].

### 4.14. MTT Assay

The color change in the 3-(4,5-dimethylthiazol-2-yl)-2,5-diphenyltetrazolium bromide (MTT, Sigma-Aldrich, Saint Louis, MO, USA) upon reduction by enzymes was measured to assess the viability of cells. Cells were treated as previously described, and an equal amount of dye was added. The cells were incubated at 37 °C until the color changed. The reaction was stopped by 10% dimethyl sulfoxide (Saiguo Biotech Co., Ltd., Guangzhou, China), and the absorbance was quantified at a wavelength of 570 nm using a microplate reader (Thermo Fisher Scientific, Waltham, MA, USA) [9].

### 4.15. Mitochondrial Potential Assay

JC-1 Mitochondrial Membrane Potential Assay Kit (Invitrogen, Carlsbad, CA, USA) was used to detect the change of MMP, following the manufacturer’s instructions. The cytofluorimetric lipophilic cationic dye, 5,5′,6,6′-tetrachloro-1,1′3,3′-tetraethylbenzamidazol-carboncyanine iodide (JC-1), can selectively enter the mitochondria and reversibly change the color from green to red as the membrane potential increased. Cells were treated as previously described, and an equal amount of JC-1 Mitochondrial Potential Probe was added. After 30 min of incubation, staining was evaluated using a Nikon Eclipse Ti-SR epifluorescence microscope (Nikon, Tokyo, Japan). In some cases, the color change was analyzed by flow cytometry (BD Biosciences, San Jose, CA, USA) [9].

### 4.16. Measurement of mtROS Production

MitoSOX Red Mitochondrial Superoxide Indicator (Invitrogen, Carlsbad, CA, USA) was used for this assay, following the manufacturer’s instructions. MitoSOX Red can selectively enter the mitochondria and produce strong red fluorescence after being oxidized by superoxide. Cells were treated as previously described, and an equal amount of MitoSOX Red Mitochondrial Superoxide Indicator was added. After 15 min of incubation, staining was evaluated using a Nikon Eclipse Ti-SR epifluorescence microscope [62].

### 4.17. Immunoblotting

Phosphorylated and total proteins derived from cells and lung homogenates were lysed in RIPA buffer. The mitochondrial protein was extracted from AMs cells using the Cytoplasmic and Mitochondrial Protein Extraction Kit (Sangon Biotech, Shanghai, China). The samples were separated by electrophoresis on 12% SDS-PAGE gels and transferred to nitrocellulose transfer membranes (Merck Millipore, CORK, IRL). Proteins were detected using primary Abs at a concentration of 1/500 or 1/1000 (all obtained from Cell Signaling Technology, Boston, MA, except IL-1β, β-actin, Affinity Biosciences, Changzhou, China) and incubated overnight. Specific interaction with the primary antibodies was detected using corresponding secondary Abs conjugated to HRP (Biosharp, Hefei, China), and signals were developed using the enhanced chemiluminescence reagents (Biosharp, Hefei, China). UVP ChemStudio (Analytik Jena, Upland, CA, USA) was used for signal detection. Gel bands were quantified by Image J software, and data were presented as means ± SD from three independent immunoblotting assays. Phosphorylated, mitochondrial, and total protein levels were determined and quantified by three successive immunoblotting membranes [63].

### 4.18. LC3 Puncta Observation

AMs cells were transfected with LC3-RFP G120A plasmids for 24 h. Cells were infected with KP at an MOI of 20 for 6 h, with or without PSPAs. Cells were observed under an LSM 510 Meta Confocal Microscope (Carl Zeiss MicroImaging, Oberkochen, Germany). LC3 puncta values were derived from 100 cells/sample [62].

### 4.19. Flow Cytometry Assay

Pyroptosis was also confirmed by flow cytometry analyses of Annexin-V and propidium iodide staining. AMs cells were treated as previously described, subsequently co-stained with annexin V-FITC/PI (KeyGEN Biotech, Nanjing, China), and analyzed by flow cytometry (BD Biosciences, San Jose, CA, USA) [64].

### 4.20. Statistical Analysis

Experiments were performed in triplicate and repeated at least three independent times. Data were shown as mean ± SD. Statistical differences were evaluated by one-way ANOVA (Tukey’s post hoc test) for multiple comparisons or by two-tailed Student’s t-test for two experimental group comparisons using GraphPad Prism 7 software (La Jolla, CA, USA). Differences were considered significant at *p* < 0.05.

## 5. Conclusions

Taken together, in this paper, PSPAs promoted mitophagy to possess considerable maintaining mitochondrial homeostasis capacity through the Nrf2 signaling pathway, thereby blocking NLRP3 inflammasome activation and inhibiting pyroptosis to reduce inflammatory responses and limiting the transmission of KP. These findings demonstrate that PSPAs could defend against KP infection and induce mitophagy as an Nrf2 activator. Moreover, these data provide evidence that mitophagy and pyroptosis regulate innate immune signaling pathways to manipulate the course of infectious diseases.

## Figures and Tables

**Table 1:** Primers used for semiquantitative PCR.

Gene	Primer
PINK1	Forward primer: ccccacaccctaacatcatc
	Reverse primer: actgggagtctgctcctcaa
Gapdh	Forward primer: caaggctgagaatgggaagc
	Reverse primer: gaagacgccagtagactcca
ND2	Forward primer: cccattccacttctgattacc
	Reverse primer: atgatagtagagttgagtagcg

## Data Availability

All the data are contained within the article.

## Supplementary Materials

The following are available online at https://www.mdpi.com/article/10.3390/ijms222111422/s1.
