# Supplementary material for: Anthocyanin Extract from Purple Sweet Potato Exacerbate Mitophagy to Ameliorate Pyroptosis in Klebsiella pneumoniae Infection
Source: Int J Mol Sci. 2021 Oct 22;22(21):11422. doi: 10.3390/ijms222111422 (PMC8583717; doi:10.3390/ijms222111422)
Supplement: Supplementary file 1 [file ijms-22-11422-s001.zip › ijms-1401851-supplementary.pdf]

Supplementary Figure and Figure legends

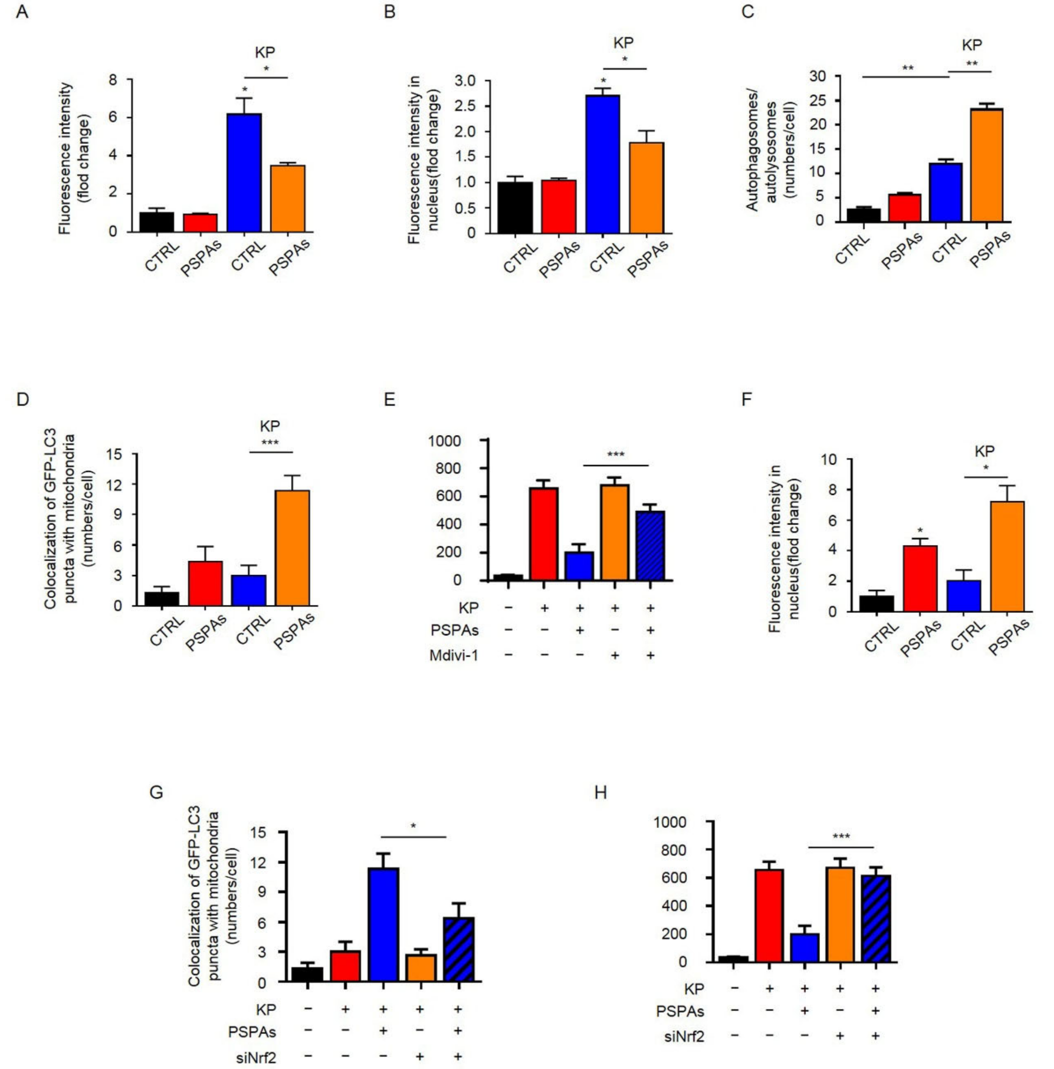

Figure S1. Columns of fluorescence intensity, autophagosomes, and number of co-localized RFP-LC3 puncta and MitoTracker Green.

(A) The quantitative analysis of fluorescence intensity in Figure 4B. **(B)** The quantitative analysis of fluorescence intensity in the nucleus in Figure 4C. **(C)** The quantification of autophagosomes in cells in Figure 5B. **(D)** The quantification of the number of co-localized RFP-LC3 puncta and MitoTracker Green was quantified in Figure 5C. **(E)** The quantitative analysis of

fluorescence intensity in Figure 5G. **(F)** The quantitative analysis of fluorescence intensity in the nucleus in Figure 6B. **(G)** The quantification of the number of co-localized RFP-LC3 puncta and MitoTracker Green was quantified in Figure 6D. **(H)** The quantitative analysis of fluorescence intensity in Figure 6H. Data were presented as mean ± SEM from at least three independent experiments. Data (mean ± SEM) are representative of three independent experiments. One-way ANOVA (Tukey's post hoc); $*P < 0.05$, $**P < 0.01$, $***P < 0.001$.
